# Multimodality Medical Image Fusion Using Clustered Dictionary Learning in Non-Subsampled Shearlet Transform

**DOI:** 10.3390/diagnostics13081395

**Published:** 2023-04-12

**Authors:** Manoj Diwakar, Prabhishek Singh, Ravinder Singh, Dilip Sisodia, Vijendra Singh, Ankur Maurya, Seifedine Kadry, Lukas Sevcik

**Affiliations:** 1 Department of Computer Science and Engineering, Graphic Era (Deemed to Be University), Dehradun 248002, Uttarakhand, India; 2 School of Computer Science Engineering and Technology, Bennett University, Greater Noida 201310, Uttar Pradesh, India; 3 Department of Computer Science and Engineering, Engineering College Ajmer, Ajmer 305025, Rajasthan, India; 4 School of Computer Science, University of Petroleum and Energy Studies, Dehradun 248007, Uttarakhand, India; 5 Department of Applied Data Science, Noroff University College, 4612 Kristiansand, Norway; 6 Artificial Intelligence Research Center (AIRC), Ajman University, Ajman 346, United Arab Emirates; 7 Department of Electrical and Computer Engineering, Lebanese American University, Byblos 13-5053, Lebanon; 8 University of Zilina, Univerzitna 1, 01026 Zilina, Slovakia

**Keywords:** clustered dictionary learning, shearlet domain, sum-modified Laplacian, medical imaging, bioelectronics

## Abstract

Imaging data fusion is becoming a bottleneck in clinical applications and translational research in medical imaging. This study aims to incorporate a novel multimodality medical image fusion technique into the shearlet domain. The proposed method uses the non-subsampled shearlet transform (NSST) to extract both low- and high-frequency image components. A novel approach is proposed for fusing low-frequency components using a modified sum-modified Laplacian (MSML)-based clustered dictionary learning technique. In the NSST domain, directed contrast can be used to fuse high-frequency coefficients. Using the inverse NSST method, a multimodal medical image is obtained. Compared to state-of-the-art fusion techniques, the proposed method provides superior edge preservation. According to performance metrics, the proposed method is shown to be approximately 10% better than existing methods in terms of standard deviation, mutual information, etc. Additionally, the proposed method produces excellent visual results regarding edge preservation, texture preservation, and more information.

## 1. Introduction

Computational methods of image processing are often used to achieve image fusion. The primary goal of image fusion is to reduce the volume of data produced with a sharp, comprehensive image that can be useful in clinical and scientific research. Compared to integrating separate modalities, synthesis outcomes between two or more modalities in a single image are more comprehensible, correct, and high quality [1]. Due to multimodality medical image fusion, a new medical image can be reconstructed via fusion algorithms. Multimodal image fusion aims to merge multiple input images into a single coherent whole. This fused image contains more medical information than individual medical images. Single sensor, multisensor, multi-view, multi-model, and multi-focus fusions are only a few types of image fusion. One sensor, part of a unified sensor fusion system, can take many sequential images from different viewpoints and fuse them into a single, unified image. Using many sensors, a system creates several distinct scene video sequences, which are combined to form a single video. Fusion imaging uses many camera angles to take images of the same scene from different vantage points [2]. By piecing together the individual photos, a whole image can be made.

By combining the images from several different medical imaging models, we can obtain a single image that is consistent throughout. When there are several input images of the same scene, each with a varied depth of focus [3,4,5], multi-focus fusion systems are used to combine the information from all of the images into one. Image fusion techniques are used for image processing in a wide variety of application fields, some of which include medical image fusion and sensor networks used for area surveillance, tracking, and environmental monitoring. These are only two examples of the many possible application areas. Under the context of this scenario, the team is required to make substantial use of diagnostic imaging data, which may include results from CT scans, MRIs, and other tests. There are situations when a single diagnostic test might not be sufficient, necessitating additional multimodal medical imaging. Even though there are a variety of different solutions available, this method of processing medical images is still widely utilized [6,7,8]. The combination of two or more medical image components results in an increased number of newly created medical images being made available for use. In this paper, a new fusion approach in the shearlet domain is presented for the purpose of integrating a wide variety of medical image types [9].

The study of image fusion has recently gained popularity due to its utility in diverse advancing fields, such as medicine, remote sensing, and defense. This technique continues to produce vital data for image fusion since it is inexpensive, resilient, and provides high-resolution images. However, obtaining crucial data for image fusion is a common and challenging issue due to the high cost of devices and the amount of blur data. 

Image fusion is the process of combining two or more images, each of which may be different from the others or identical, to create a new image that incorporates features from each original image. This new image should keep maximum information from the original images while also minimizing any artifacts that may have been introduced during the fusion process, as is the case in many practical applications [10]. The fundamental objective of fusion is to create a single high-resolution image from a collection of lower-resolution ones. Sharp images are necessary for diagnosing diseases, such as coronary artery disease (CAD), which develops when the heart does not receive adequate oxygen. In addition, neurologists play an important role in the prognosis of brain tumor conditions; hence, image fusion is used to analyze brain scans from different modalities. Each researcher’s motivations might make image fusion an intriguing and novel problem. Satellite imaging, medical imaging, aviation, the detection of concealed weapons, the use of digital cameras for battlefield monitoring and situational awareness, the tracking of targets with surveillance cameras, and the authentication of individuals in the geo-informatics industry are just a few of the many modern applications of image fusion [11]. Reading the literature has numerous benefits, such as dictionary learning, cluster analysis, sum-modified Laplacian (SML), and contrast-based fusion. 

In the present study, low-frequency fusion sub-bands with a new SR method use coincidental instances that allow DTCWT and SR simultaneously. Patches in the source image were found using structural similarities, which were then classified and grouped into clusters. All the condensed sub-dictionaries in the cluster are compressed and merged to form an adaptive, clustered, and condensed sub-dictionary (ACCD). The fusion algorithm forms a one-of-a-kind algorithm known as the modified sum-modified Laplacian (MSML), which is based on the LARS algorithm and synthesizes sparse coefficients from the synthetic sparse vectors that are formed using the fusion algorithm. An example of this is the employment of a sub-band fusion approach, which combines the usage of the high-frequency maximal complete ruling, and consistency affirmation working together in an instance. More information about the patient’s health can be gleaned through the fusion of data from many medical imaging modalities. The evidence synthesis in radiographic images is one example of the multimodal approach to a medical diagnosis that recent advances in the field have favored. To better understand the patient’s blood flow and metabolic rate, it is necessary to segment all the medical images depending on their relatively inadequate functional image spatial resolution. The physical structure is assumed to obtain a reasonably high spatial resolution.

With these motivations, a new multimodality medical image fusion method is proposed. The major contributions of the paper include:

  i. A dictionary learning method based on cluster analysis is introduced in low-frequency sub-band fusion. In this technique, structural image patch attributes are pooled and mathematically connected to increase computation efficiency;
 ii. For low sub-band fusion, the modified sum-modified Laplacian (MSML) constructs artificially sparse vectors by employing saliency features to calculate low-frequency sub-band local features;
iii. A directive contrast-based fusion is introduced by calculating the local facts of high-frequency sub-band MSML.

The rest of this paper is organized as: In Section 2, related work is discussed. Section 3 shows the methods that are utilized in the proposed work. In Section 4, the proposed work is discussed. Section 5 shows the result and the discussion. Finally, Section 6 draws conclusions. 

## 2. Related Work

Zhang et al. [12] proposed a multimodality medical image fusion method where multiscale morphology gradient-weighted local energy and a visual saliency map are used to improve the results of existing state-of-the-art methods. The results are good in terms of the statistical methods and visual appearance. However, the contrast of the fused image is not up to the mark for many complex images. Ramlal et al. [13] introduced a method using a hybrid combination of non-subsampled contourlet transform and stationary wavelet transform for medical image fusion. The results are also good regarding visual appearance and performance metrics. However, due to more multiple transforms, the computation cost is increased. To combine medical images from different modalities, Dogra et al. [14] proposed utilizing guided filters and image statistics in the multidirectional shearlet transform domain. Multimodality medical picture fusion was proposed by Ullah et al. [15], who suggested using local features fuzzy sets in conjunction with a novel sum-modified Laplacian in a non-subsampled shearlet transform domain. The non-subsampled shearlet transform and the activity measure were proposed by Huang et al. [16] as a method for optimizing information gain during picture fusion. Shearlet-domain-based fusions produce good results generally, but their lack of contrast in high-texture photos is suboptimal. Multimodality medical image fusion was proposed by Liu et al. [17], using an image decomposition framework, non-subsampled shearlet transformation, and a weighted fusion function. Mehta et al. [18] proposed using a guided filter in the NSCT domain to achieve more comprehensive informatics outcomes in multimodality medical image fusion. Though the guided filter produces respectable outcomes overall, its performance falls short for images with a dense texture in terms of edge retention.

Contrast-based fusion rules are also employed for fusion purposes, and local energy is given to the reactor. Because of this, edges are more reliably found and stabilized when the decomposition approach is employed. In [19], a new layer-based fusion approach considers layer differences by separating the base and detail layers while using saliency characteristics that seek coincidences. Using the characteristics’ ability to highlight important regions of relevance, crisp and smooth fusion outcomes may be achieved with minimal effort. Over the last year, SR-based fusion approaches have suffered a significant drop in popularity in the multisensor image fusion sector. This SR-based fusion technology is only successful if the dictionary is overflowing with components and the best-in-class fusion algorithm is constructed. DCT, DWT, Gabor, and Ridgelet are some of the sparse fusion methods often used [20,21,22]. Another image that is frequently used is that of dictionary learning. Dictionary entries become significantly more difficult when working with images with complicated structures. An intelligent learner merges all the patches from the input images using a heuristic dictionary, proving his or her intelligence. Because the choice of a dictionary is crucial in SR, researchers have referred to the image patch clustering technique. Despite this, a large number of images remain unsolved [23,24,25]. The computation costs of dictionary learning [26,27,28,29,30] and sparse coding are higher than wavelet-based fusion methods. 

The use of a suitable fusion rule enables the successful synthesis of a sparse artificial set of coefficients, which is observed in the following cases: A dictionary-based learning approach requires two parameters to be effective for individuals who do not desire to calculate words in advance: how long it takes to build the data and how many steps the learner repeats in the process of creating the data. Because the ideal learning parameter in classical techniques, such as the K-SVD, is decided by the set of rules, it is difficult to manage learning time. Furthermore, sparse coding may incur additional expenses since it may need an evaluation cost less than the input datas’ size. Sparse representation: the number of patches increases in proportion to the size of the input data. On the other hand, SR-based fusion techniques have historically met difficulties because the rules used are rarely relevant in the current temporal context of the experiment. Fusion algorithms, which are commonly used to find visible images in an infrared image algorithm, produce images that are visible in the IR results when applied to the IR results.

The importance of medical imaging, in both medical research and clinical practice with an intent to achieve high image quality, is increasing and demands representation or simulation. In certain situations, the complete spectrum structure of digital image processing can aid in medical diagnosis. Radiologists can diagnose organs or illnesses effectively, with a combination of images of the organs or diseases involved. It is noted that the type and model of the instruments used in medical imaging also restrict their ability to offer such information. The presence of vital organs or living tissues is referred to as “heterogeneity” in medical imaging. The differences in size and shape can occur even when the same modality is used to gather the data due to factors such as the object’s shape, internal structure, or even just the fact that separate images of the same patient were acquired at various times. The boundary between foreground and background cannot be erased in the study of biological anatomy. The outcomes of automatic medical image analysis are dependent on several factors. Photo blending has been proven to enhance image quality drastically. The error- and redundancy-free multimodality medical image fusion technique aims to improve image quality [31,32,33,34,35,36].

Wadhwa et al. suggested a mechanism for predicting the lockdown period to be implemented to successfully contain the spread of COVID-19 in India [37]. Four methods were employed to create an epidemic alarm system, including Random Forest Regression, Decision Tree Regression, Support Vector Regression, and Multiple Linear Regression [38]. Dhaka et al. [39] analyzed the differences between the stationary wavelet transform (SWT) and the discrete wavelet transform (DWT) for different applications and found SWT outperforms DWT. According to a study by Dhaundiyal [40], a novel SWT-based multimodality fusion approach was presented for medical image fusion. In this method, the source images are first decomposed into an approximation layer (coarse layer) and a detail layer using the SWT scheme and then the Fuzzy Local Information C-Means Clustering (FLICM) and local contrast fusion approach are applied to the distinct layers to counteract the blurring effect, maintain sensitivity, and preserve quality evaluation. The suggested approach [41] uses a non-subsampled shearlet transform (NSST) to extract low and high-frequency components from input images. Low-frequency components are fused using a co-occurrence filter (CoF), and a unique process is employed to deconstruct and merge the base layers and detail layers using the local extrema (LE) approach. Sum-modified Laplacian (SML) is used to fuse the high-frequency coefficients in an edge-preserving image fusion approach [41].

## 3. Preliminaries

This section presents an overview of the methods that are used in the proposed work. Some of the main methods are discussed here in the subsections below.

### 3.1. Non-Subsampled Shearlet Transform (NSST)

The NSST is not only a practical instrument for multiscale geometric research because of its amazing ability to discover linear singularities but also a correct description of the 2-D sparse method. This is because of its success in detecting linear singularities. The shift-invariance and anisotropic direction selectivity of the discrete wavelet transform are signature features that set it apart from other wavelet transform types. The discrete wavelet transform is an especially useful tool for deciding the location of point-wise singularities. Because it uses a non-subsampled Laplacian pyramid filter, the NSST can perform multiscale directional localization thanks to this implementation choice. It outperforms NSCT in several crucial areas, including productivity, flexibility, and stability against orientation changes, to name a few of these categories. Through NSST, images are decomposed into two major parts, (i) low-frequency components and (ii) high-frequency components. These low- and high-frequency components provide the features of the images, which can be utilized here for multimodality medical image fusion. 

### 3.2. Clustered Dictionary Learning

In this method, the clusters-based dictionary is generated by finding the local features in terms of patches, which can be further used for image fusion. The previously mentioned approach shows that the patch P_k_ ∈ P lies next to edge cluster Ce if its activity level is more than a threshold. Suppose the patch’s activity level is lower than the lowest threshold, it refers to a smooth cluster ${𝒞}_{𝒮}$, except for when it refers to texture cluster ${𝒞}_{𝓉}$. This procedure is repeated continuously until all patches in the joint patch set P are arranged. At last, every cluster in the set of clusters ${𝒞}_{𝒮}$ is data trained in the online dictionary-based learning algorithm, and the compressed sub-dictionaries ${𝒟}_{ℯ},{𝒟}_{𝓉}$, and ${𝒟}_{𝒮}$ are acquired. All the acquired sub-dictionaries are integrated to make a dictionary D. Create the ${𝒞}_{ℯ},{𝒞}_{𝓉},{𝒞}_{𝒮}$-clustered sub-dictionaries and then create the 𝒟-clustered combined dictionary where each of the clusters is trained to apply the ODL algorithm to attain the resulting sub-dictionaries ${𝒟}_{ℯ},{𝒟}_{𝓉},\;\mathrm{and}\;{𝒟}_{𝒮},\;\mathrm{respectively},$ and join each sub-dictionary to make the final dictionary, such as $𝒟=\left\{{𝒟}_{ℯ},{𝒟}_{𝓉},{𝒟}_{𝒮}\right\}$, where ${𝒟}_{ℯ},\;{𝒟}_{𝓉},\;\;\mathrm{and}\;{𝒟}_{𝒮}$ are the sub-dictionaries of edge cluster, texture cluster, and set of clusters, respectively.

### 3.3. Visual Saliency Features

To extract saliency characteristics, the largest symmetric surround saliency method is employed (MSS). The following is the method that must be used to implement the largest symmetric surround saliency (MSS) method, as shown in Equation (1).

*P*(*i*, *j*) = ||*P*_1_(*i*, *j*) − *P*_2_(*i*, *j*)|| (1)

where *P*(*i*, *j*) are the saliency features, *£*(*i*, *j*) is the average pixel values of all CIELAB, and || || is the L2 norm. The average pixel values of all CIELAB are obtained as shown in Equation (2).

(2) $$P(i,\;j)\;=\;\frac{1}{r}\left(\sum_{x=i-m}^{i+m}\sum_{y=j-n}^{j+n}\left[P\left(x,y\right)\right]\right)$$

where *m* = *min*(*i*, *w*–*i*), *n* = *min*(*j*, *h*–*j*), *r* = (2*i* + 1)(2*j* + 1), *w* and *h* are the width and height of the image, respectively.

## 4. Proposed Methodology

**Figure 1:**
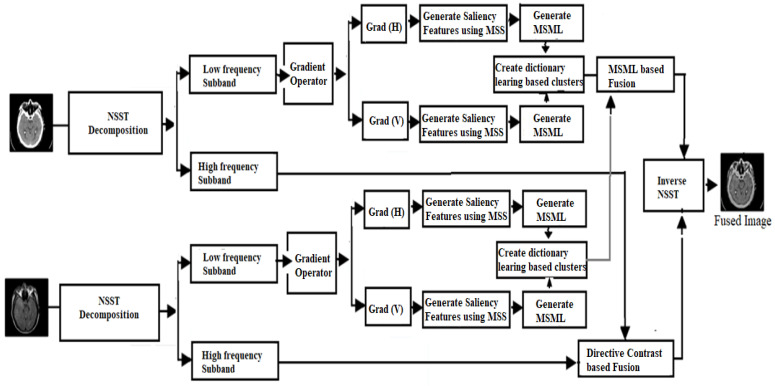
Multimodality medical image fusion proposed framework.

In the proposed methodology, two different modality images are utilized as input images. Initially, NSST is performed over both input images to obtain low- and high-frequency components. Over the low-frequency components of both input images, a gradient operator is applied to obtain horizontal and vertical direction for extracting detailed features. Over these features, saliency features are obtained by utilizing the concept of MSS. Over these saliency features, a modified SML operation is introduced. These features are further clustered by performing dictionary-based learning method. Using modified SML operation, fusion operation is performed on both dictionary-learning-based clusters. On the other side, high-frequency components are processed using directive contrast-based fusion. Finally, inverse NSST is performed over both modified low- and high-frequency components. In proposed work, a dictionary-based learning algorithm is first defined and then the complete fusion substructure of the concept and the technique connected to the fusion algorithm on sub-band images are described. The fundamental objective of this study is to develop a compact, well-organized over-completion dictionary with the optimal structure and high computational efficiency to compete with existing dictionary-based learning approaches. To demonstrate the efficacy of the new approach to dictionary creation, a clustering-based learning mechanism for categorizing input image patches with a geometrically similar structure is used. Therefore, the following features of the input pictures can be maintained and exploited for accurate segmentation. The borders, textures, and smooth areas of an image are the key image components that may alter the overall texture; hence, it is focused on in the present study. In any given image, the details at the image’s edges and textures stand out the most. Edges are perceived differently depending on the smoothness of the component, but they still blend into the background when viewed by a person. The steps of the proposed algorithm are shown in Figure 1.

Step 1 (NSST decomposition): Perform *NSST* decomposition on input images with parameters c = 1 and d = 8 to obtain low- and high-frequency components on both input multimodal medical images, as shown in Equation (3).

(3) $$\left[L_{f1}^{NSST},\;H_{f1}^{NSST}\right]=NSST\left(A_{i,j}\right)\;\mathrm{and}\;\left[L_{f2}^{NSST},\;H_{f2}^{NSST}\right]=NSST\left(B_{i,j}\right)$$

Step 2 (Low sub-band fusion): The gradient operator is used to obtain horizontal and vertical orientation across the low-frequency components of both input images in order to extract finer information. On top of these components, the idea of maximum saliency (MSS) is used to obtain saliency attributes. A refined SML technique is introduced over these prominent indicators. We then use a dictionary-based learning approach to further categorize these traits. We perform a fusion operation on both clusters based on dictionary learning using a modified *SML* procedure. Perform the below sub-steps to obtain a low sub-band fused image.

(a) Find the gradient information *GA* and *GB* in horizontal and vertical directions from both input images;
(b) Estimate modified Laplacian (*ML*), as shown in Equation (4);
  (4) $$ℳℒ\left(i,j\right)\;=\;abs\left(P\left(i,j\right)*Grad_{ℋ}\left(i,j\right)\right)+abs\left(P\left(i,j\right)*Grad_{V}\left(i,j\right)\right)$$
(c) Develop *MSML* by adding the ML as shown in Equation (5);
  (5) $$MSML\left({𝓅}_{𝓀}\right)=\sum_{i=1}^{𝓃}\sum_{j=1}^{𝓃}ML\left(i,j\right)$$
  where 𝓃×𝓃 is the size of ${𝓅}_{k}$;
(d) Acquire the ${𝒞}_{ℯ},{𝒞}_{𝓉},{𝒞}_{𝒮}$ clusters using *MSML*:
  (i) Separate the source images $I_{A}\;\mathrm{and}\;I_{B}$ into 𝓃×𝓃 patches, $P_{A}\;\mathrm{and}\;P_{B}$, respectively;
  (ii) Combine $P_{A}\;\mathrm{and}\;P_{B}$ to make a joint patch set $P=\left\{P_{A},P_{B}\right\}$;
  (iii) Search the MSML for every ${𝓅}_{𝓀}\in P$;
  (iv) Fix the thresholds $TH_{1},TH_{2}$ by utilizing as shown in Equations (6) and (7):
    (6) $$TH_{1}=0.13\;*\;max\left(MSML\left({𝓅}_{h}\right)\right)$$
    (7) $$0.07\;*\;\max\left(MSML\left({𝓅}_{h}\right)\right)$$
(e) Perform the equation below to make the $C_{ℯ},\;C_{𝓉},\;C_{S}$ clusters. The categorization approach is described, as shown in Equation (8);
  (8) $$C_{J}=\left\{\begin{array}{c} C_{ℯ},\;if\;MSML\;\left({𝓅}_{h}\right)\geq TH_{1}\\ C_{𝓉},\;if\;TH_{1}>MSML\left({𝓅}_{h}\right)\geq TH_{2}\\ C_{S},\;if\;TH_{2}>MSML\left({𝓅}_{h}\right) \end{array}\right\}$$
(f) The sum-modified-Laplacian (*SML*) is a technique that has proven effective in the field of medical picture fusion. When applied to the altered image, fusion rules based on a larger *SML* always lead to either information loss in the fused spatial domain or image distortion. New filters, the average filter, and the median filter, are available in the latest version of *SML*, which is utilized for medical picture fusion. *MSML* is the main computation to evaluate all activity levels of the image patch. It elaborates on the small information, the image constraint. Increasing the value gives more details as it exists. Suppose $MSML\;\left(𝒾;L_{A}\right)$ and $MSML\left(𝒾;L_{B}\right)$ represent the ${𝒾}^{\mathrm{th}}$ patch’s modified *SML* of low-frequency sub-images $L_{A}\;\mathrm{and}\;L_{B}$, the recommended fusion approach is described, as shown in Equation (9):
  (9) $$a_{L_{F}}^{𝒾}=\left\{\begin{array}{c} a_{L_{A}}^{𝒾},\;if\;MSML\left(𝒾;L_{A}\right)\geq MSML\left(𝒾;L_{B}\right)\\ a_{L_{B}}^{𝒾},\;otherwise \end{array}\right\}$$
  where $V_{L_{F}}^{𝒾}=Da_{L_{F}}^{𝒾}+{𝓂}_{L_{F}}^{𝒾}$ and the fusion mean value ${𝓂}_{L_{F}}^{𝒾}$ is followed by Equation (10),
  (10) $${𝓂}_{L_{F}}^{𝒾}=\left\{\begin{array}{c} {𝓂}_{L_{A}}^{𝒾},\;if\;a_{L_{F}}^{𝒾}=a_{L_{A}}^{𝒾}\\ {𝓂}_{L_{B}}^{𝒾},\;otherwise \end{array}\right\}$$

Step 3 (High sub-band fusion): The coefficients show that the sub-images with higher frequencies often have information from the source image. Moreover, because noise is usually caused by high frequencies, it can mess up calculations for fusion, which can lead to wrong sharpness values and hurt the quality of the fusion process. To illustrate these results, a new set of criteria based on the use of directed contrast has been made. According to the step-by-step approach, the following is an explanation of the complete operation.

(a) Estimate the directive contrast ($D_{L}\left(i,j\right))$ of *NSST* high-frequency coefficients using low sub-band coefficients as shown in Equations (11) and (12):
  (11) $$\;\;D_{LA}\left(i,j\right)=\left\{\begin{array}{c} \frac{MSML\left(A\left(i,j\right)\right)}{A\left(i,j\right)}\;\;\;if\;A\left(i,j\right)>0\\ MSML\left(A\left(i,j\right)\right)\;\;\;Otherwise \end{array}\right.$$
  Similarly,
  (12) $$\;\;D_{LB}\left(i,j\right)=\left\{\begin{array}{c} \frac{MSML\left(B\left(i,j\right)\right)}{B\left(i,j\right)}\;\;\;if\;B\left(i,j\right)>0\\ MSML\left(B\left(i,j\right)\right)\;\;\;Otherwise \end{array}\right.$$
(b) Apply the following fusion rule to the high-frequency coefficients ($Hf\left(i,j\right)$) as shown in Equation (13):
  (13) $$Hf\left(i,j\right)=\left\{\begin{array}{c} Hf_{A}\left(i,j\right)\;\;\;if\;D_{LA}>D_{LB}\\ Hf_{B}\left(i,j\right)\;\;\;Otherwise \end{array}\right.$$

Step 4: Follow the below for obtaining a fused image using inverse *NSST* as shown in Equation (14):

(14) $$R=NSST^{-1}\left(Lf,Hf\right)$$

## 5. Experimental Results

Using the software MATLAB Version 9.4 (R2018a: India), the experimental evaluation was completed. The proposed methodology for multimodality medical image fusion was performed. 

### 5.1. Dataset

The analysis was carried out on the entire collection of 210 medical images that were coupled together. The images were obtained from a public access database Atlas (http://www.med.harvard.edu/AANLIB/home.html (accessed on 22 May 2022)) [36]. The multimodal imaging modalities that are frequently used are CT scanning and magnetic resonance imaging (MRI). The complex make-up of human tissue delivers information that is more precise and detailed than ever before. The ability of CT scans to supply highly correct anatomical reconstructions makes them useful not only for diagnosis but also for treatment. When seen at an oblique angle, more of the inner workings of an organ are clear. On the other hand, bone, soft tissue, and lung are more beneficial for studying the skeletal and connective tissue components. One of the methods used to obtain a better understanding of the human body is the use of windows that allow looking through bone, soft tissue, or the lungs, i.e., SPECT. SPECT images are used rather often in the field of CT imaging. These multimodality medical images are further used in our experimental analysis. All the images used for experimental results had a resolution of 512 × 512. If the resolution size of both input image is not the same, preprocessing should be applied to obtain the same resolution of the input images. However, we tested all experiments on the same resolutions of both input images.

**Figure 2:**
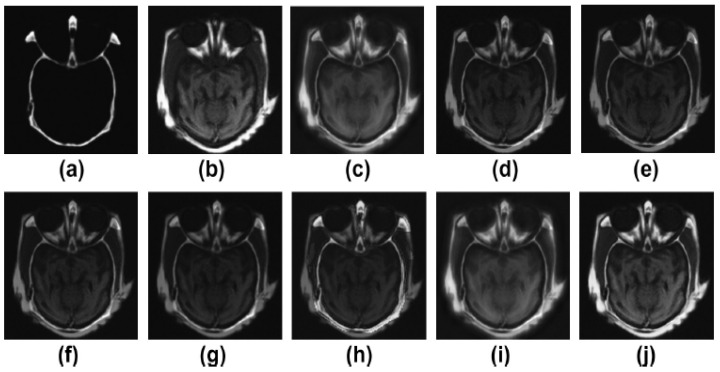
Results of multimodality medical image fusion; (**a**) input multimodality medical image 1; (**b**) input multimodality medical image 2; (**c**) Zhang et al. [12]; (**d**) Ramlal et al. [13]; (**e**) Dogra et al. [14]; (**f**) Ullah et al. [15]; (**g**) Huang et al. [16]; (**h**) Liu et al. [17]; (**i**) Mehta et al. [18]; (**j**) proposed method.

**Figure 3:**
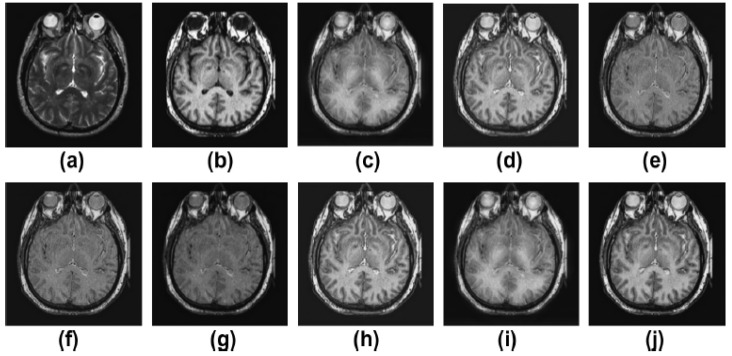
Results of multimodality medical image fusion; (**a**) input multimodality medical image 1; (**b**) input multimodality medical image 2; (**c**) Zhang et al. [12]; (**d**) Ramlal et al. [13]; (**e**) Dogra et al. [14]; (**f**) Ullah et al. [15]; (**g**) Huang et al. [16]; (**h**) Liu et al. [17]; (**i**) Mehta et al. [18]; (**j**) proposed method.

**Figure 4:**
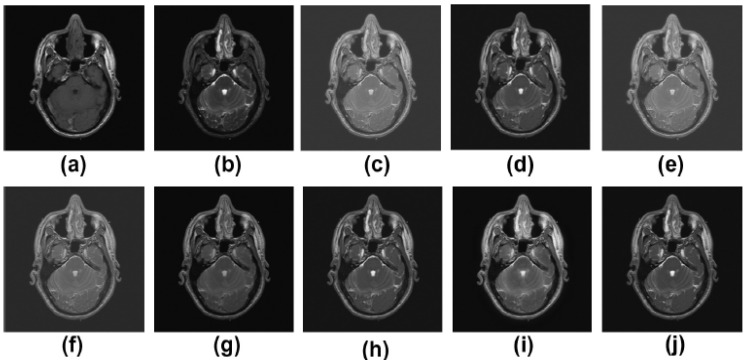
Results of multimodality medical image fusion; (**a**) input multimodality medical image 1; (**b**) input multimodality medical image 2; (**c**) Zhang et al. [12]; (**d**) Ramlal et al. [13]; (**e**) Dogra et al. [14]; (**f**) Ullah et al. [15]; (**g**) Huang et al. [16]; (**h**) Liu et al. [17]; (**i**) Mehta et al. [18]; (**j**) proposed method.

**Figure 5:**
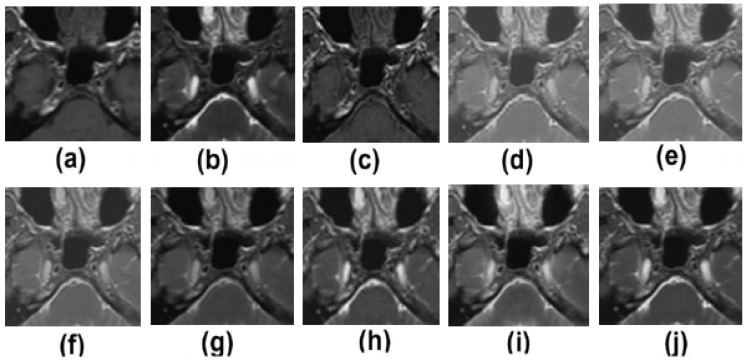
Zoomed results of multimodality medical image fusion; (**a**) input multimodality medical image 1; (**b**) input multimodality medical image 2; (**c**) Zhang et al. [12]; (**d**) Ramlal et al. [13]; (**e**) Dogra et al. [14]; (**f**) Ullah et al. [15]; (**g**) Huang et al. [16]; (**h**) Liu et al. [17]; (**i**) Mehta et al. [18]; (**j**) proposed method.

Pairs of medical images are available in the public database (http://www.med.harvard.edu/AANLIB/home.html (accessed on 22 May 2022)), and they include modalities including computed tomography (CT) and magnetic resonance imaging (MRI). There are numerous multimodality effects seen in Figure 2, Figure 3, Figure 4 and Figure 5.

### 5.2. Results and Discussion

The proposed methodology is compared with recently proposed methods, such as those of Zhang et al. [12], Ramlal et al. [13], Dogra et al. [14], Ullah et al. [15], Huang et al. [16], Liu et al. [17], and Mehta et al. [18]. 

Figure 2a,b are the two input multimodalities CT and MRI. Figure 2c–j are the results of Zhang et al. [12], Ramlal et al. [13], Dogra et al. [14], Ullah et al. [15], Huang et al. [16], Liu et al. [17], Mehta et al. [18], and the proposed method, respectively. In Figure 2, the results are good in terms of edge preservation and providing more informatics clinical details. In this respect, the results of [12] are good, but the textures in homogenous regions are not effectively preserved. Similarly, the results of [13] are also not effectively preserved in terms of contrast and brightness. The results of [14,17] are well preserved in all the details, but in highly textured regions, the results are not excellent. The results of [15,16,18] are good but the high-textured details are not satisfactory. However, in comparison to others, the proposed method gives the best results in terms of sharpness, smoothness, texture preservation, and more informatic clinical details.

Figure 3a,b display both the MR-T2 image and the SPET image. The findings of [12,13,14,15,16,17,18] as well as the proposed approach are presented in Figure 3c–j, respectively. In Figure 3, the outcomes are favorable in terms of edge preservation and added clinical data gleaned through informatics. While [12] achieves outstanding results overall, it does a less than stellar job of preserving textures in areas with high homogeneity. The brightness and contrast of the outputs of [13] are likewise not kept very well. The outcomes of [14,17] are great in low-textured areas and good in high-textured areas, respectively. The results of [15,16,18] are satisfactory, but the high-textured details are lacking. Sharpness, smoothness, texture preservation, and more informatic clinical features are all improved upon using the proposed strategy. On the other hand, in contrast to earlier methods, the one that is proposed yields the best results in terms of sharpness, smoothness, the preservation of texture, and additional informatics clinical features.

The MR-T2 image as well as the SPET image are presented in Figure 4a,b, respectively. The results of Zhang et al. [12], Ramlal et al. [13], Dogra et al. [14], Ullah et al. [15], Huang et al. [16], Liu et al. [17], Mehta et al. [18] are provided in Figure 4c–j, respectively, along with the suggested methodology. Figure 4 demonstrates that the outcomes are positive in terms of edge preservation as well as new clinical data obtained through informatics. The method that has been offered, on the other hand, in contrast to those that have been used in the past, produces the best results in terms of sharpness, smoothness, the preservation of texture, and extra informatics clinical aspects. The results of [12] are good in this instance, but the textures in homogeneous regions are not particularly well preserved. The results from [13] are similarly poorly preserved in terms of contrast and brightness. The results from [14,17] are excellent in that all of the details are preserved, but the results are less than perfect in highly textured regions. Although [15,16,18] produce good results, the high-textured detail is not particularly satisfactory. The proposed method, however, produces the best results when compared to other approaches in terms of sharpness, smoothness, texture preservation, and more clinical informatics information.

Figure 5a,b show zoomed-in regions of input multimodality medical images. Figure 5c–j show the results of Zhang et al. [12], Ramlal et al. [13], Dogra et al. [14], Ullah et al. [15], Huang et al. [16], Liu et al. [17], Mehta et al. [18] as well as the suggested technique, respectively. Figure 5 shows that the outcomes in terms of edge preservation and additional clinical data collected through informatics are both positive. The method presented, on the other hand, produces the best results in terms of sharpness, smoothness, texture preservation, and extra informatics clinical characteristics as compared to previous methods. The findings of [12] are satisfactory in this regard; nonetheless, the textures in the homogenous zones are not kept remarkably. The contrast and brightness of the results of [13] are likewise not adequately preserved in the same way. The outputs of [14,17] do a good job of preserving all of the features, but in areas with a lot of texture, their performance is less than stellar. The results of [15,16,18] are satisfactory; however, the particulars of the high-textured results are not particularly outstanding. On the other hand, in contrast to previous methods, the one that is proposed yields the best results in terms of sharpness, smoothness, the preservation of texture, and additional informatics clinical features.

Visual results were not sufficient for the resulting analysis; hence, the results of the existing methods were tested and evaluated using performance metrics. To check the accuracy of the existing methods, some parameters were used, such as ${\mathrm{MI}}_{\mathrm{AB},F},{\;Q}_{\mathrm{AB},F},\;\mathrm{and}\;\mathrm{BSSIM}$. The results were tested over 80 pairs of medical images and the average values are shown in Table 1. From Table 1, it can be analyzed that the transform domain approaches give better outcomes. The bold values in Table 1 show the best performance metric values for different image datasets.

## 6. Conclusions

A diagnostic image analysis based on multimodality is presented in the present study. Advanced human data should be sensitive to better contrast (high), pixel density, edge detail, contrast focus, view dependencies, fusion device edge, and texture detection.

The proposed method gives better results in terms of visual results such as smoothness and sharpness in high-textured images. Other than the visual results, performance metrics are also evaluated where the values of the performance metrics show better results in comparison to existing methods. The study discusses several forms of errors in imaging data. Moreover, it showcases the lack of noise and the improvement in the information presented in the fused image and compares the data obtained for calculation from the original image. The findings suggest that current transform domain methods have better outcomes than using other spatial domain structures. The performance metrics also prove that in addition to visual effects, techniques using transform domain strategies provide enhanced results compared to analogous spatial domain schemes.

## Figures and Tables

**Table 1:** The Comparative analysis in terms of performance metrics.

Parameter	Dataset	Zhang et al. [12]	Ramlal et al. [13]	Dogra et al. [14]	Ullah et al. [15]	Huang et al. [16]	Liu et al. [17]	Mehta et al. [18]	Proposed Method
Mutual information (MI)	#1	2.1298	2.7849	3.0512	3.4810	3.3952	3.2967	3.1719	3.4917
	#2	2.7972	3.1168	2.4757	2.5788	2.2534	3.1270	3.5670	3.7710
	#3	2.5610	2.6513	2.8315	2.4103	2.4124	2.7109	2.3709	2.8709
	#4	2.1268	2.3103	2.2330	2.6150	2.2612	2.7120	2.1720	2.8710
	#5	2.2111	2.2171	2.3212	2.1167	2.1019	2.1418	2.1178	2.6418
Standard deviation (SD)	#1	66.2122	81.0191	75.9053	84.2526	82.8310	81.0198	82.0498	85.0563
	#2	58.5118	72.0111	72.8118	75.1325	76.4587	77.1798	78.1448	78.7798
	#3	55.2596	71.2195	71.1124	71.2723	71.1187	71.2272	71.2710	72.2350
	#4	58.5555	72.5422	71.1446	73.3550	74.2444	75.0320	72.2320	75.1180
	#5	67.8141	66.1515	69.0115	71.5219	72.2217	71.8761	72.8716	73.2276
QAB/F	#1	0.5101	0.5115	0.5202	0.5113	0.5218	0.5103	0.5301	0.5397
	#2	0.5183	0.5140	0.5178	0.5218	0.5187	0.5251	0.5211	0.5288
	#3	0.5919	0.6151	0.6281	0.6271	0.6171	0.6311	0.6351	0.6398
	#4	0.6141	0.6117	0.6220	0.6217	0.6428	0.6363	0.6151	0.6486
	#5	0.6171	0.6312	0.6151	0.6222	0.6123	0.6454	0.6352	0.6510
Spatial frequency (SF)	#1	23.1212	27.7511	25.5710	26.8186	26.714	27.0110	27.5504	27.9822
	#2	21.1113	22.7833	22.6141	21.6113	21.5422	22.4123	22.7233	22.8123
	#3	19.0926	21.1813	21.0111	20.1818	20.0718	20.0019	21.0019	21.3319
	#4	17.3556	18.2313	18.1112	20.1122	19.4554	20.0013	20.1313	20.2923
	#5	20.0961	18.8329	21.4140	18.3431	19.9129	18.2390	19.0120	21.4190
Mean	#1	49.3249	58.2346	53.8543	57.1209	56.0238	57.5120	57.5121	58.5350
	#2	44.1433	51.7246	47.4440	51.3356	52.1270	53.8219	51.3409	53.9609
	#3	41.3453	41.1233	39.1753	41.0125	39.1241	38.1240	38.2134	42.7970
	#4	40.4680	41.3643	39.1233	41.3430	38.3252	41.1122	41.1414	41.8324
	#5	33.1282	36.8872	35.9921	34.4503	35.5453	33.4657	36.4457	37.0057

## Data Availability

Not applicable.

## Abbreviations

NSST	Non-subsampled shearlet transform
MSML	Modified sum-modified Laplacian
CAD	Coronary artery disease
ACCD	Adaptive, clustered, and condensed sub-dictionary
SWT	Stationary wavelet transform
DWT	Discrete wavelet transform
FLICM	Fuzzy Local Information C-Means Clustering
SML	Sum-modified Laplacian
CoF	Co-occurrence filter
LE	Local extrema
NSCT	Non-subsampled contourlet transform
ML	Modified Laplacian
MRI	Magnetic resonance imaging
CT	Computed tomography
